# In vitro shoot organogenesis from *Eucalyptus* sp. leaf explants

**DOI:** 10.1186/1753-6561-5-S7-P134

**Published:** 2011-09-13

**Authors:** Juliana Degenhardt-Goldbach, Marguerite Quoirin, Surya Buss, Yohana de Oliveira, Luziane Franciscon, Isabel Gerhardt

**Affiliations:** 1 Embrapa Forestry, Brazilian Agricultural Research Corporation, Colombo, Paraná, 83411-000, Brazil; 2 UFPR, Curitiba, Paraná, Brazil; 3 Graduating student UFPR, Brazil; 4 PhD student UFPR, Brazil

## Background

*In vitro* organogenesis is one of the key techniques associated with genetic transformation, as it determines the successful regeneration of transgenic plants after co-cultivation with bacteria. Therefore, the development of efficient regeneration protocols is the most critical step in developing genetic transformation. Protocols have been developed for several species of eucalyptus. In most of the studies cotyledon, hypocotyl and leaf segments of plants cultivated *in vitro *are used as explants [1]. Several eucalyptus functional genomics projects have used *Populus* species as a model for it counts with well established regeneration and transformation protocols. However, the use of *Eucalyptus* clones as model plants could be more adequate, if clones with high regeneration rates as those obtained for *Populus *could be found. The aim of this study was to evaluate several variables in the *in vitro* organogenesis from leaves of an *Eucalyptus* sp. clone maintained *in vitro* at Embrapa Forestry.

## Methods

The experiments were performed at the Laboratory of Tissue Culture of Embrapa Forestry, Colombo, PR. Leaf explants were collected from *in vitro* grown plants maintained on MS medium [2] containing 30 g L^-1^sucrose, 0.88 µM BAP, 0.05 µM NAA, 0.5 g L^-1^and 7 PVP g L^-1^ agar. The youngest leaves were cut longitudinally and placed with the adaxial side facing the media. In the first experiment, the effect of 0.5 µM thidiazuron (TDZ) was compared with different concentrations of zeatin (2.28, 4.56, 9.12 and 13.68 µM). The plant growth regulators were added to the basic medium, composed of MS salts with half of nitrogen concentration (N/2), supplemented with vitamins of Morel and Wetmore, 30 g L^-1^sucrose, 0.1 g L^-1^myoinositol, 0.1 µMNAA and 7 g L^-1^agar. The second experiment compared the effect of MS salts N/2, WPM [3] and B5 [4] on the same basic medium described above, containing 0.5 µM TDZ. In the third experiment the effect of different concentrations of TDZ (0.25, 0.5, 0.75, 1 and 2 µM) added to the basic medium, replacing the MS N/2 by WPM was evaluated. The explants were kept in growth chamber with controlled temperature at 23 ± 2 °C in the dark for four weeks, and then transferred to the same medium after 2 weeks. After this period the explants were transferred to basal medium with WPM, 20 g L^-1^sucrose, 0.1 g L^-1^myo-inositol, 5 µM BAP, 0.05 µM NAA and 7 g L^-1^agar and placed under a photoperiod of 16 hours. The pH of all media was adjusted to 5.8 before autoclaving. After two months explants oxidation, callus formation, shoot formation in explants with callus and number of shoots per explants with callus were evaluated. Each treatment consisted of five Petri dishes with 10 explants. Data were analyzed by an analysis of variance. Comparisons between treatments were made by orthogonal contrasts.

## Results and conclusions

In the first experiment, there was no callus formation or shoot regeneration on media containing zeatin in any tested concentration. These results contradict those found by [5] who observed shoot formation on leaf explants on medium containing the combination of zeatin and NAA. However, these authors have used salts formulations other than MS. In the treatment with TDZ, 10% of explants regenerated shoots. The number of shoots per callus ranged from one to more than 10. In the second experiment, B5 medium was not favorable for shoot induction. All explants oxidized, although they formed small calli. In the comparison between WPM and MS N/2, in both media 6% of explants regenerated shoots. However, the WPM showed less oxidation than MS N/2. In the third experiment, where several concentrations of TDZ were compared, the number of explants with callus induction was lower in treatment with 0.25 µM TDZ (83%). In all other treatments all explants formed callus. The number of explants with shoots ranged from 7.5% in the treatment with 0.75 µM to 14% in treatments with 0.25 and 0.50 µM TDZ. Treatments with 1 and 2 µM TDZ showed 11% and 12% of calli with shoots, respectively. In *E. globulus*[*6*] observed buds induction on hypocotyls when placed on medium containing 0.05 µM TDZ and 5 µM NAA or 0.2 µM 2,4-D in. In *E. saligna* 40% of the calli induced on cotyledon explants regenerated shoots on medium containing 1 µM TDZ and 0.1 µM NAA [7]. Among the media tested, the WPM medium containing 0.5 µM TDZ and 0.1 µM NAA was the most suitable for *in vitro* organogenesis of the *Eucalyptus* sp. clone tested. However, regeneration rates (14%) are still low and further research is needed.
